# Towards Real-time Metabolic Profiling of Cancer with Hyperpolarized Succinate

**DOI:** 10.4172/2155-9937.1000123

**Published:** 2016-01-11

**Authors:** Niki M. Zacharias, Christopher R. McCullough, Shawn Wagner, Napapon Sailasuta, Henry R. Chan, Youngbok Lee, Jingzhe Hu, William H. Perman, Cameron Henneberg, Brian D. Ross, Pratip Bhattacharya

**Affiliations:** 1 Department of Cancer Systems Imaging, University of Texas MD Anderson Cancer Center, Houston, USA; 2 Department of Bioengineering, Rice University, 6100 Main Street, Houston, USA; 3 Department of Biomedical Sciences, Cedars-Sinai, Los Angeles, USA; 4 Enhanced Magnetic Resonance Laboratories, Huntington Medical Research Institutes, Pasadena, USA; 5 Department of Applied Chemistry, Hanyang University, Ansan, Korea; 6 Department of Radiology, School of Medicine Saint Louis University, St. Louis, USA

**Keywords:** hyperpolarization, metabolic imaging, succinate, magnetic resonance spectroscopy, krebs cycle

## Abstract

**Purpose:**

The energy-yielding mitochondrial Krebs cycle has been shown in many cancers and other diseases to be inhibited or mutated. In most cells, the Krebs cycle with oxidative phosphorylation generates approximately 90% of the adenosine triphosphate in the cell. We designed and hyperpolarized carbon-13 labeled succinate (SUC) and its derivative diethyl succinate (DES) to interrogate the Krebs cycle in real-time in cancer animal models.

**Procedures:**

Using Parahydrogen Induced Polarization (PHIP), we generated hyperpolarized SUC and DES by hydrogenating their respective fumarate precursors. DES and SUC metabolism was studied in five cancer allograft animal models: breast (4T1), Renal Cell Carcinoma (RENCA), colon (CT26), lymphoma NSO, and lymphoma A20.

**Results:**

The extent of hyperpolarization was 8 ± 2% for SUC and 2.1 ± 0.6% for DES. The metabolism of DES and SUC in the Krebs cycle could be followed in animals 5 s after tail vein injection. The biodistribution of the compounds was observed using ^13^C FISP imaging. We observed significant differences in uptake and conversion of both compounds in different cell types both *in vivo* and *in vitro*.

**Conclusion:**

With hyperpolarized DES and SUC, we are able to meet many of the requirements for a useable *in vivo* metabolic imaging compound – high polarization, relatively long T_1_ values, low toxicity and high water solubility. However, succinate and its derivative DES are metabolized robustly by RENCA but not by the other cancer models. Our results underscore the heterogeneity of cancer cells and the role cellular uptake plays in hyperpolarized metabolic spectroscopy.

## Introduction

Metabolism is fundamental to the ability of the cell to replicate and survive. Metabolic changes are now considered one of the hallmarks of cancer [1]. Changes in metabolism (type and concentration of metabolites) are increasingly realized as biomarkers and reporters of phenotype [2]. Cancer cells up-regulate glycolysis in a process known as the Warburg effect [3]. In addition, cancer can utilize a variety of compounds and pathways for energy metabolism and for replication in the generation of fatty acids and nucleic acids [4,5]. Recent publications have revealed that the Krebs Cycle and Oxidative Phosphorylation is not inhibited in many cancer cells, as previously thought, but that these cells utilize other compounds than glucose to feed these metabolic pathways (such as glutamine, acetate) [2,6]. In addition, several genetic mutations in three Krebs cycle enzymes, succinate dehydrogenase, fumarate hydratase, and isocitrate dehydrogenase, have been revealed to be oncogenic [7–9] and several targeted therapies have been generated that specifically interact with the Krebs cycle and Oxidative Phosphorylation [10–12]. The ability to monitor the *in vivo* flux rate of the Krebs cycle would allow for the efficacy of these compounds to be determined and potentially allow for patients prior to treatment to be sub-divided as responders and non-responders. We describe our efforts in generating a hyperpolarized metabolic imaging agent to determine the *in vivo* flux rate of the Krebs cycle in cancer bearing animals.

Magnetic Resonance Imaging (MRI) and Magnetic Resonance Spectroscopy (MRS) of hyperpolarized reagents enable real-time imaging of metabolic alterations. Hyperpolarization allows for >10,000 fold sensitivity enhancement over Boltzmann polarization. The polarization (signal enhancement) can be retained by the metabolites of the hyperpolarized molecule [13–17]. The most widely used methods for hyperpolarization of organic compounds are Dynamic Nuclear Polarization (DNP) and Parahydrogen Induced Polarization (PHIP). Unlike Positron Emission Tomography (PET), the process of hyperpolarization is non-radioactive. Hyperpolarized metabolic imaging can also be non-toxic, minimally-invasive, and can provide physiologic and anatomic information at any stage of disease evaluation, be it screening, diagnosis, treatment, or surveillance.

PHIP is a novel technique, whereby the altered spin equilibrium from para enriched hydrogen is transferred to a chemical of interest. This causes a magnetic response far beyond the Boltzmann polarization as in conventional Nuclear Magnetic Resonance (NMR) [13,16]. During the hydrogenation reaction, a radiofrequency pulse is applied to transfer the signal enhancement from parahydrogen to the carbon-13 atom. The radiofrequency heteronuclear pulse is generated using the coupling constants between the carbon-13 atom and the attached hydrogens as described by Golman [18]. Each sample of hyperpolarized diethyl succinate or succinate requires 4 seconds of polarization. A new sample can be generated every three to four minutes.

There are several requirements to developing an excellent and broad utility hyperpolarized metabolic imaging agent – the compound needs to be (1) highly polarizable (2) have low toxicity and high solubility in water (3) a long spin-lattice relaxation time (T_1_) (4) needs to be taken up by cell within the time frame of polarization (5) needs to be metabolized to metabolic products within the cell in the time frame of polarization. For steps 4 and 5, uptake and metabolism of most carbon-13 labeled hyperpolarized metabolic imaging agents need to occur in the minute(s) time frame. For the compound to have any translational potential, the metabolic products of the hyperpolarized agent must also have low toxicity, long T_1_ values (10 s or longer), and unique chemical resonances from the parent compound (> 2 ppm). Many compounds can be polarized but very few have all the characteristics above for translational development. With hyperpolarized DES and SUC, we are able to meet most of the needs for a clinically relevant metabolic imaging compound – high polarization (8 ± 2% SUC [10] and 2.1 ± 0.6% DES [11]), relatively long T_1_ values (43.7 ± 0.3 s at pH 8.5 and 9.6 ± 0.2 s at pH 3.5 SUC, 54 ± 2 s DES), low toxicity and high water solubility. In this report, we describe the first *in vivo* studies of SUC and DES in tumor bearing animals.

## Materials and Methods

### Hydrogenation and polarization

Hyperpolarized DES was generated by hydrogenation of diethyl 1-^13^C 2,3-d_2_ fumarate to diethyl 1-^13^C-2,3-d_2_ succinate in aqueous solution using a bisphosphine rhodium catalyst [19]. The final pH of solution was 6. Hyperpolarized SUC was generated by adding 1-^13^C fumarate-d_2_ (Cambridge Isotope Laboratories, Andover, MA) to the rhodium catalyst. The resulting mixture contained 1–3 mM fumarate and 2.0–2.5 mM catalyst concentrations in 50 mM pH 2.9 (or pH 10.5) phosphate buffer [17,20].

With both agents, the aqueous mixture of catalyst and molecular precursor was prepared fresh, prior to each hyperpolarization procedure. The completed solution of precursor and catalyst was drawn into a 20 mL plastic syringe for injection of the desired amount of imaging reagent precursor for each experiment (3.5 mL unless otherwise noted) into the reaction chamber of the polarizer. Hydrogenation was performed in our home built reaction chamber [21]. The reaction chamber was heated and filled with parahydrogen gas (DES 60°C, 12 bar H_2_: SUC 62°C, 10 bar H_2_). When prepared in these proportions, hydrogenation of the precursor with parahydrogen gas was carried to completion and no residual precursor fumarate or diethyl fumarate was detected by ^13^C NMR. During the hydrogenation reaction a radiofrequency transfer pulse was applied to transfer polarization to carbon-13 labeled atom. Aliquots of 0.5 ml of hyperpolarized, hydrogenated ^13^C reagent were then injected into the mouse tail vein. (See Supplemental Methods for Further Discussion on Animal Models)

### MRI and MRS

All MRI and MRS of animals and imaging phantoms (plastic MR compatible object for internal control employed in MR experiments) were performed in an animal 4.7 T MR (Bruker Avance, Bruker AG, Germany) horizontal bore scanner. For hyperpolarized studies, MRS and MRI were performed after complete injection of 0.5 ml of hyperpolarized SUC or DES. On average the injection took 15–20 s.

### *In vivo* experiments with hyperpolarized SUC

For all SUC experiments two successive injections (0.5 ml 10 to 30 mM SUC via tail vein) were utilized to increase the hyperpolarized SUC signal and concentration in the mice.

#### ^13^C MRS with SUC

The typical magnetic resonance spectroscopy acquisition consisted of 12 averages acquired every 5.6 seconds with a bandwidth of 25000 Hz and 1096 points and 32 repetitions for a total time of 3 minutes. A fast gradient imaging technique produced ^13^C imaging of tumors in 3.5 seconds after injecting hyperpolarized succinate via tail vein catheter. A 25 mm multi-turn ^1^H-^13^C homemade loop coil was fitted around the tumor. Five slices were obtained with a 0.5 × 1 × 3.2 mm^3^ voxel resolution. As a result of the high signal produced by the hyperpolarized succinate and relatively long T^1^, we were able to produce continual images every 3.5 seconds over the course of one minute with a low flip angle. Two ^13^C spectroscopy experiments were performed interlaced with the imaging experiments. Eight low flip angle (~10°) spectra were acquired with 15 second delays between acquisitions. In total, each mouse received between two and five injections over the course of the experiment before it was euthanized.

For further information on MR sequences used to monitor hyperpolarized SUC metabolism and biodistribution see Supplemental Methods.

### *In vivo* experiments with hyperpolarized DES

For all DES experiments, ^13^C MRS and ^13^C MRI was taken after tail vein injection of 0.5 ml of 20 mM hyperpolarized DES.

#### DES ^13^C MRI imaging

A full body ^1^H/^13^C dual tuned volume coil (Doty Scientific Inc., Columbia, SC) was used in all experiments to allow co-registration of carbon hyperpolarized images with mouse ^1^H MRI anatomic images. Mice were placed prone in the volume coil. Proton coronal or axial imaging was done with a FOV and slice thickness corresponding to the ^13^C image using a modified Bruker MSME sequence. Carbon-13 imaging was done using Bruker TRUE FISP sequence (TR = 3.3 ms, TE = 1.6 ms, 4 averages, 32 × 32 matrix and bandwidth 52083 Hz). Flip angles of 80, 60 or 40 degrees were used in the carbon-13 imaging sequence. Coronal imaging was performed using a FOV of 6 or 7 cm, one to two slices with dimensions 15.2 mm, and the center slice was always selected to be the same as proton images. Images were then converted into false color in Paravision 3.0.2 Software. The image was then overlaid on the proton image. No ^13^C MRI image was seen if hyperpolarized DES was not injected.

Magnetic field homogeneity was adjusted using single voxel proton MRS (PRESS) data acquisition approach and the voxel (0.7 × 0.7 × 0.7 cm^3^) of interest was placed within the subcutaneous tumor. The unsuppressed water signal less than 15 Hz was routinely obtained. Shimming was essential for peak resolution in ^13^C MRS experiments. ^13^C Chemical Shift Imaging (CSI) was performed using an 8 × 8 or 12 × 12 matrix with elliptical sampling using a 30° pulse with a FOV of 2.64 cm to 5 cm, TR of 200 ms and slice thickness of 9.56 cm either using the volume or solenoid coil. Each CSI scan took approximately 13 s. CSI was processed using 3DiCSI software (Columbia University, Qui Zhao) with autophasing, 5 Hz line broadening, and centering the DES signal to 176.4 ppm. Hyperpolarized phantoms and carbon-13 labeled acetate phantoms were used to verify acquisition parameters. (See Supplemental Methods for Diethyl Succinate Tissue Culture Experiments)

## Results

### Hyperpolarization and spin lattice relaxation values (T_1_)

**Figure 1:**
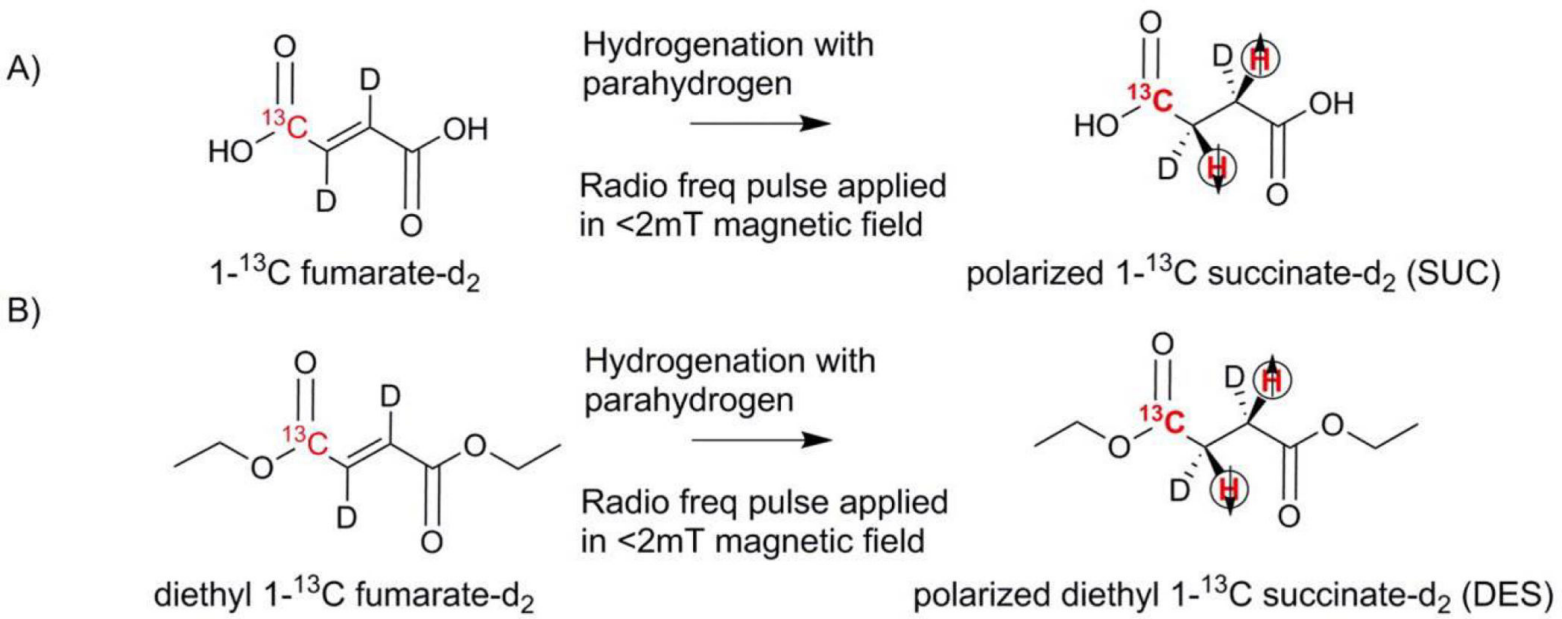
Chemistry of hyperpolarizaton of 1-^13^C succinate and diethyl 1-^13^C succinate via PHIP (a) Cis molecular addition of parahydrogen to 1-^13^C-fumaric acid-d_2_ to produce 1-^13^C-succinic acid-d_2_. (b) Cis molecular addition of parahydrogen to diethyl 1-^13^C-fumaric acid-d_2_ to produce diethyl 1-^13^C-succinic acid-d_2_. Radio frequency pulses are applied to transfer the spin order inside a 1.8 mT electromagnet followed by ejection of hyperpolarized 1-^13^C-succinic acid-d_2_. The entire process is automated in the polarizer as described in ref. [17,21].

For SUC, ^13^C fumarate-d_2_ is hydrogenated to 1-^13^C succinate-d_2_ (Figure 1A) and hyperpolarized to a level of 8 ± 2% by PHIP, (signal enhancement ~20,000 fold compared to Boltzmann polarization). Hydrogenation and hyperpolarization of diethyl 1-^13^C fumarate-d_2_ (Figure 1B) yields DES with a polarization level of 2 ± 0.6% (signal enhancement 5000 fold compared to Boltzmann polarization). For SUC, the hydrogenation has to occur at low pH (3.5) or high pH (8.5) to have well defined coupling constants for either completely charged or uncharged succinate [20].

The hyperpolarized signal enhancement of SUC and DES relaxes over time and is dependent on the T_1_ (spin lattice relaxation time) of the carbon-13 labeled atom. The T_1_ for hyperpolarized SUC and DES was determined by measuring the decay of the polarized carbon signal over time on our 4.7 T MR scanner. The T_1_ of the labeled carbonyl in DES is 38 ± 4 s *in vivo* and 54 ± 2 s in a phantom (in 9:1 water to D_2_O solution of 20 mM hyperpolarized DES) [19]. Experimentally determined *in vivo* longitudinal relaxation times (T_1_) of SUC (surface coil over mouse tumor) was 43.7 ± 0.3 s and 9.6 ± 0.2 s at pH 8.5 and 3.5, respectively, at 4.7 T. These T_1_ values correspond well to other hyperpolarized agents in the literature [22]. With the ability to measure approxiametly 5 * T_1_ in most of our experirments, we are able to track the metabolism of DES and SUC for one to two minutes depending on the size of the flip angle used to the detect hyperpolarized signal.

### Hyperpolarized SUC metabolism in five tumor types

Acidic pH 3 – 4.5 (N=40) and alkaline pH 8.5 (N=39) injections of hyperpolarized SUC (Table 1) were evaluated in five tumor types: CT26 colon, 4T1 breast, NSO lymphoma, lymphoma A20 and RENCA renal carcinoma. A longer duration of hyperpolarized ^13^C signals at alkaline pH was seen and is attributed to the prolonged T_1_. Each ^13^C MRS is taken from two injections of 0.5 ml of hyperpolarized ^13^C succinate with the total time for each hyperpolarized experiment being 370 s. While hyperpolarized ^13^C succinate was detected in all five of the tumor-types tested (colon 26, breast 4T1, lymphoma NSO, lymphoma A20, RENCA), hyperpolarized metabolites were observed only in lymphoma A20 and RENCA tumors, with significant metabolism only observed in the RENCA tumors.

**Figure 2:**
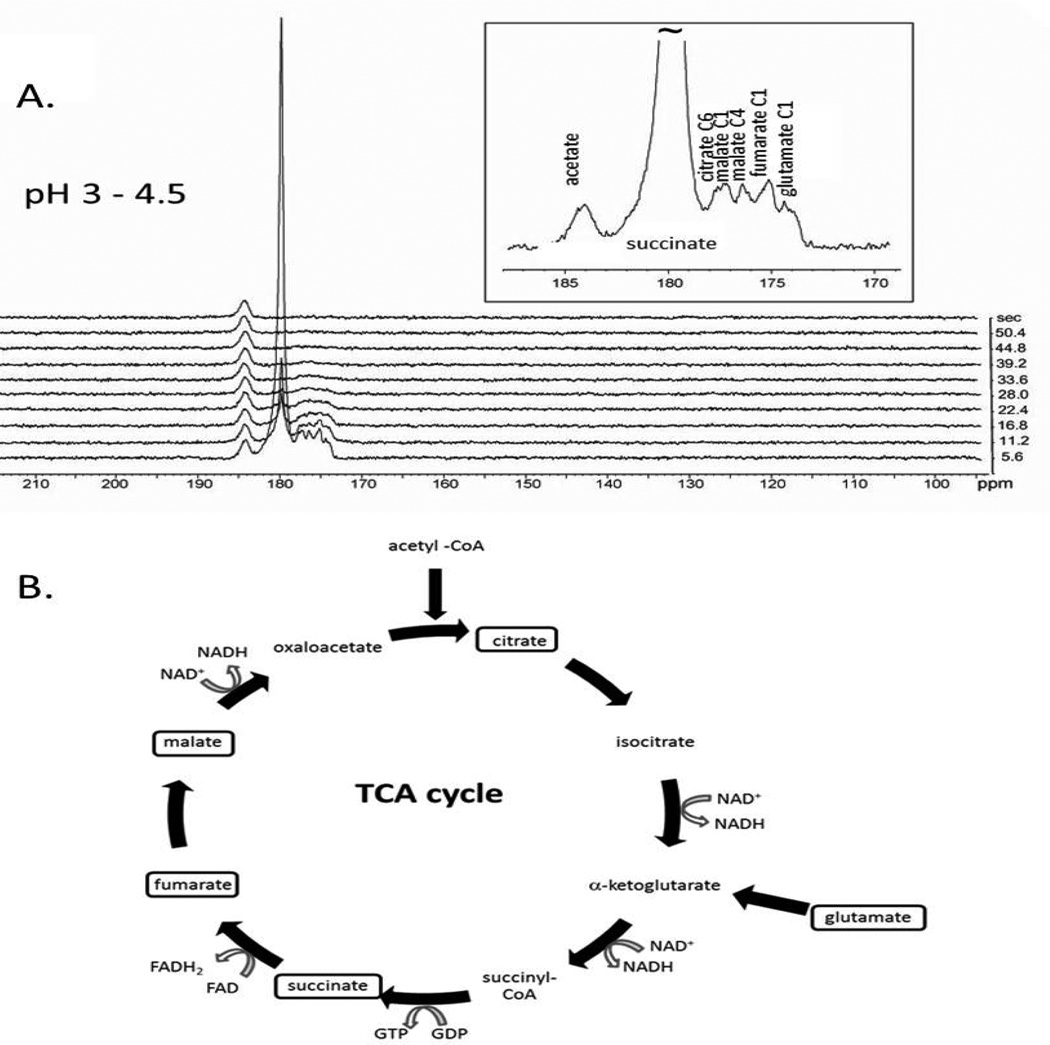
Real time detection of Krebs cycle metabolism in a subcutaneous RENCA of a living mouse using hyperpolarized ^13^C MRS. The mouse received 10 µmoles hyperpolarized succinate by tail vein injection. Metabolic products from hyperpolarized ^13^C SUC (the largest peak) were detected in the tumor within 10 seconds of the injection of the hyperpolarized substance via the tail vein (pH 3 – 4.5) and persisted through 28 secs. Using the 3 M ^13^C acetate phantom (non-hyperpolarized) as a reference, the four distinct metabolites are assigned to malate C1, malate C4, citrate C6, fumarate C1 and glutamate C1. This experimental result was confirmed in six animals. The pattern of the resonances as well as the tentative assignments by chemical shift, strongly suggests that hyperpolarized ^13^C succinate is metabolized *in vivo* and that metabolites of succinate within Krebs cycle retain a significant fraction of hyperpolarized signal. B. The corresponding Krebs cycle of oxidative cellular metabolism in RENCA tumors is illustrated. The metabolite pools of implanted tumor which become enriched from exogenous ^13^C succinate within the T_1_ of hyperpolarization are indicated in boxes.

Real time detection of Krebs cycle metabolism was observed in subcutaneous RENCA tumor bearing mice using hyperpolarized ^13^C SUC (Figure 2). Metabolic products from hyperpolarized ^13^C SUC (the largest peak) were detected in the tumor within 10 seconds of the injection of the hyperpolarized substance via the tail vein (pH 3 – 4.5) and persisted through 28 sec. Note, the ^13^C chemical shift of succinate carboxyl group is pH dependent ([20], unpublished work in the laboratory) (180 ppm at pH 3.5). Using the 3M ^13^C acetate phantom (non-hyperpolarized) as a reference, the four distinct metabolites were assigned as malate C1, malate C4, citrate C6, fumarate C1 and glutamate C1 (Figure 2A: inset). Since the input metabolite is 1-^13^C succinate, only citrate C6 and glutamate C1 is observed in RENCA, consistent with enrichment through reactions of the mitochondrial Krebs cycle (Figure 2B). This experimental result was confirmed in six animals. The pattern of the resonances as well as the tentative assignments by chemical shift, strongly suggests that ^13^C succinate hyperpolarized by the PHIP technique is metabolized *in vivo* and that metabolites of succinate retain a significant fraction of the hyperpolarized ^13^C nuclei through three or more enzyme catalyzed biochemical reactions.

**Figure 3:**
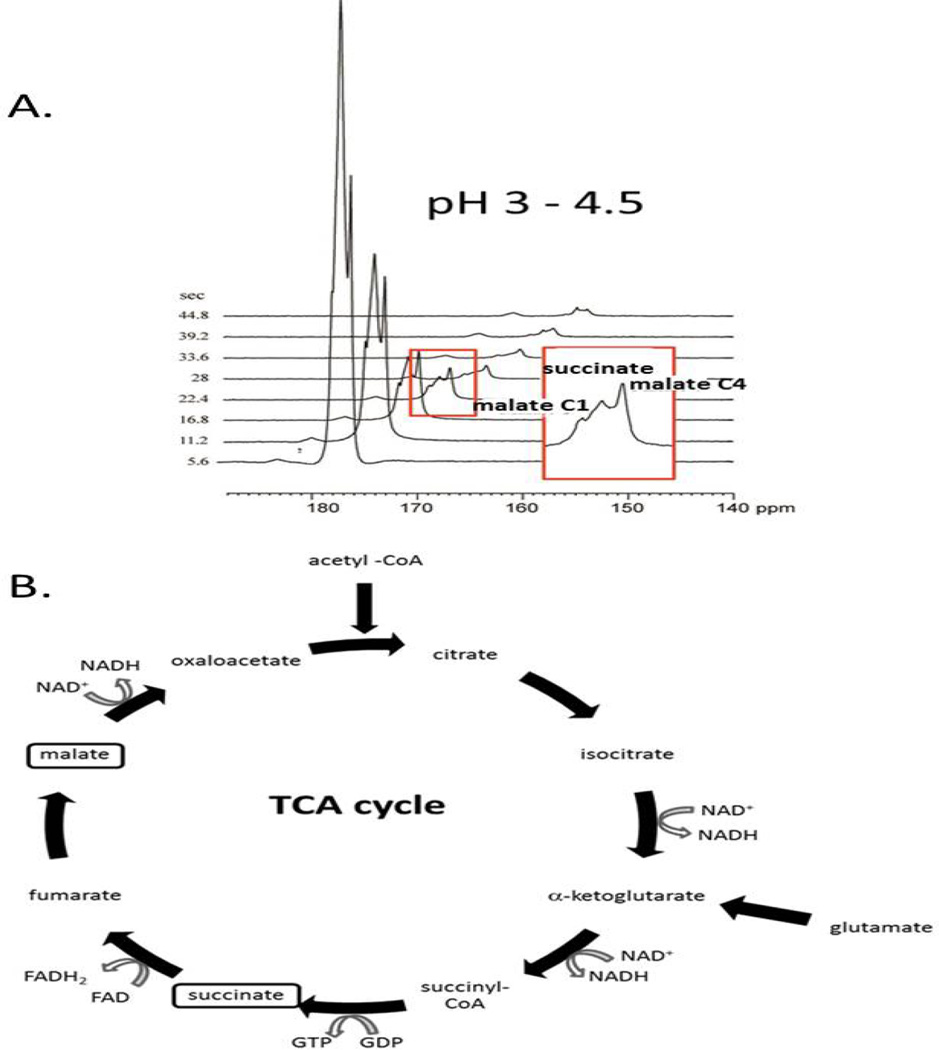
Real time detection of hyperpolarized ^13^C succinate and its metabolites in lymphoma A20 in a living mouse. The mouse received 10 µmoles hyperpolarized SUC by tail vein injection. A. Metabolic products from hyperpolarized ^13^C SUC (the largest peak) were detected in the tumor within 10 seconds of the injection of hyperpolarized substrate (pH 3 – 4.5) and persisted for 45 seconds. Using the 3 M ^13^C acetate phantom (non-hyperpolarized) as chemical shift reference, metabolites are defined as C1 and C4 malate. B. The corresponding Krebs cycle of oxidative tumor metabolism in lymphoma A20 is illustrated. Krebs cycle metabolite pools which reproducibly become enriched from exogenous ^13^C succinate within the T_1_ of hyperpolarization are indicated in boxes and differ markedly from those in RENCA.

Limited metabolism of hyperpolarized SUC was seen in lymphoma A20 (Figure 3). The mouse received 10 µmoles of hyperpolarized SUC by tail vein injection. Metabolic products from hyperpolarized SUC (the largest peak) were detected in the tumor within 10 seconds of the injection of hyperpolarized substrate (pH 3 – 4.5) and persisted for 45 seconds (Figure 3A). Using the 3M ^13^C acetate phantom (nonhyperpolarized) as a chemical shift reference, metabolites are defined as malate C1 and malate C4. An identical experiment performed at pH 8.5 showed an almost identical metabolic profile of hyperpolarized succinate in a subcutaneous lymphoma A20 tumor (Supplemental Figure 1). The hyperpolarized signal of succinate is longer lasting at pH 8.5 consistent with our experimentally observed *in vivo* T_1_ values.

### Estimation of Krebs cycle rate using hyperpolarized succinate

**Figure 4:**
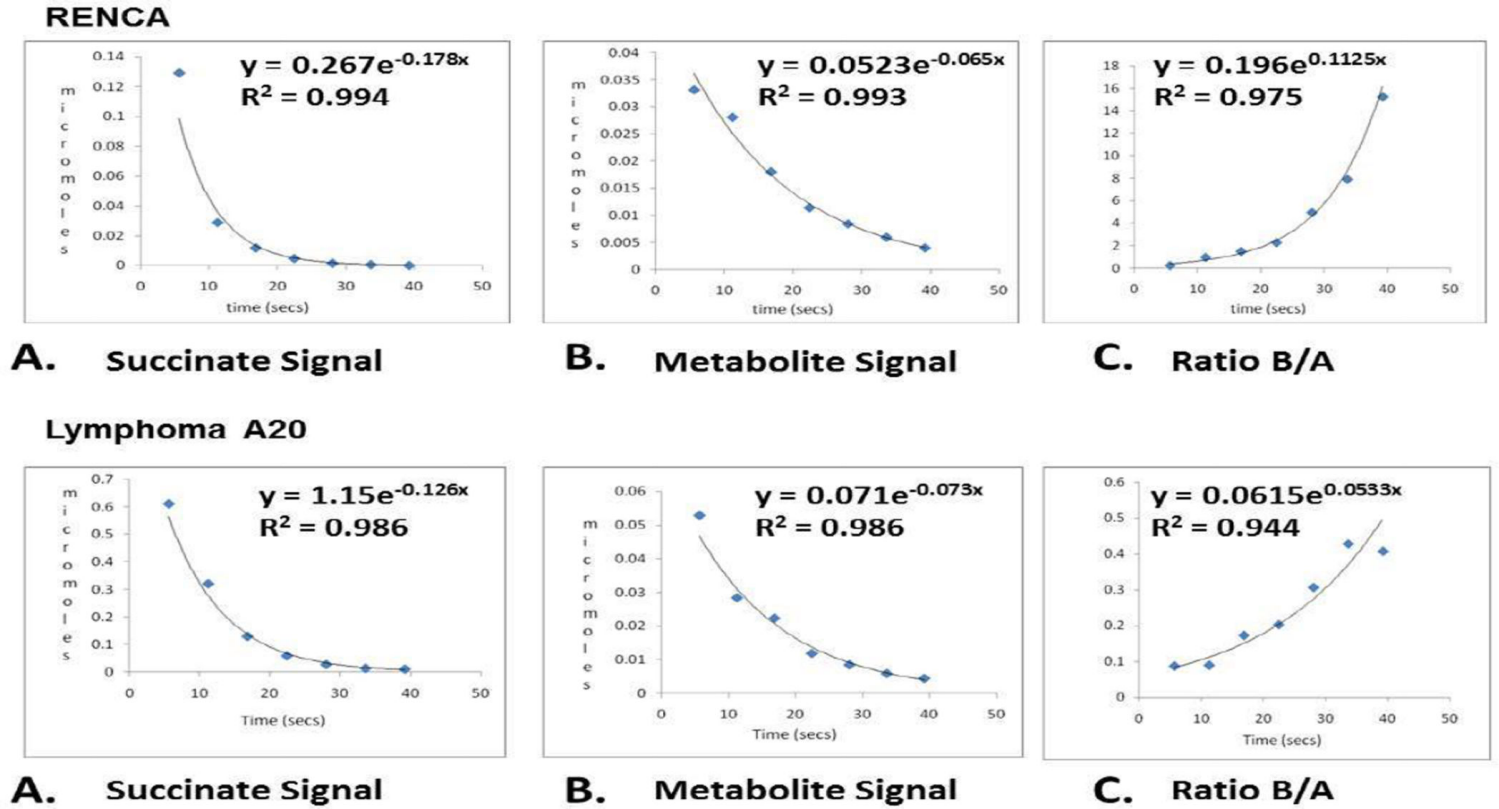
Curve A is the observable signal and normalized concentration of hyperpolarized succinate over time. Curve B is the observable signal and normalized concentration of hyperpolarized metabolites over time. Each curve is the composite of T_1_ decay of hyperpolarization, metabolic transformation into hyperpolarized ^13^C products and losses as a result of radiofrequency pulses necessary for their *in vivo* detection. The ratio malate/succinate (M/S) or total metabolite/succinate when plotted against time (Curve C) provides an estimate of the minimum rate of accumulation of hyperpolarized ^13^C into metabolite(s) in the tumor. For details, see text. Partial Krebs cycle rate for RENCA (succinate to citrate/glutamate) was estimated at 5 µmoles/minute/gram (upper curves) and for lymphoma A20 (succinate to malate) at 0.7 µmoles/minute/gram (lower curves).

Another major challenge in hyperpolarized metabolic imaging is determining the “true” flux rate of hyperpolarized agents to their metabolic products. The rate is dependent on the uptake of the agent into tissue/cells and the metabolic transformation in the cell. In addition, both the hyperpolarized imaging agent and its metabolites’ observable signal(s) are decaying over time (T_1_) and a percentage of magnetization is utilized for each observation. Usually the imaging agent and its metabolites have different T_1_ values and these values are usually lower in an *in vivo* setting. This makes “true” flux quantitation difficult. We utilized a rather simple approach to get an approximate value for the Krebs cycle “rate” of accumulation of hyperpolarized ^13^C metabolites as shown in Figure 4. This simple approach does not take into account the T_1_ decay of hyperpolarization for succinate or its metabolites or the amount of magnetization used for each measurement. We tried to minimize these changes by using a 10° flip angle to obtain the data and, because the increased magnetization is on the carboxylic acid of succinate and its metabolites, all of the compounds should have similar T_1_ values. We did not take into account the amount of agent in the blood versus the tissue but blood flow was comparable in both models (gadolinium T_1_ imaging data not shown).

Curve A is the observable signal of hyperpolarized succinate in RENCA and Lymphoma A20 mice over time. Curve B is the observable signal of hyperpolarized metabolites over time (Figure 4). The area under half of the succinate resonance and half of the malate peak in Lymphoma A20 and half the peak area of the sum total of the overlapping metabolite peaks in RENCA were used to determine the observable *in vivo* signal. These values were then compared to the integrated value of the 3M ^13^C acetate reference used in all experiments. The reference is doped with Gd-DPTA and was therefore, assumed to have complete relaxation between acquisitions. The signal per mole of acetate was determined and divided by the enhancement factor (difference in spin population for 5% polarization divided by the Boltzmann difference in spin population). Multiplying this value by the signal observed for succinate and metabolites resulted in an estimate of the moles present of each compound. The ratio of the change in concentration of metabolites (Curve B) to change in the concentration of hyperpolarized succinate (Curve A) when plotted against time provides a rough estimate of the minimum rate of accumulation of hyperpolarized ^13^C into malate/metabolites in the tumor through the Krebs cycle. Based on these decay curves, it is estimated that net flux through the portion of Krebs cycle described by the metabolic products is ~5 µmoles/minute/gram tumor in RENCA (succinate to citrate/ glutamate) and 0.7 µmoles/minute/gram tumor in lymphoma A20 (succinate to malate only).

### *In vivo* experiments with hyperpolarized DES

**Figure 5:**
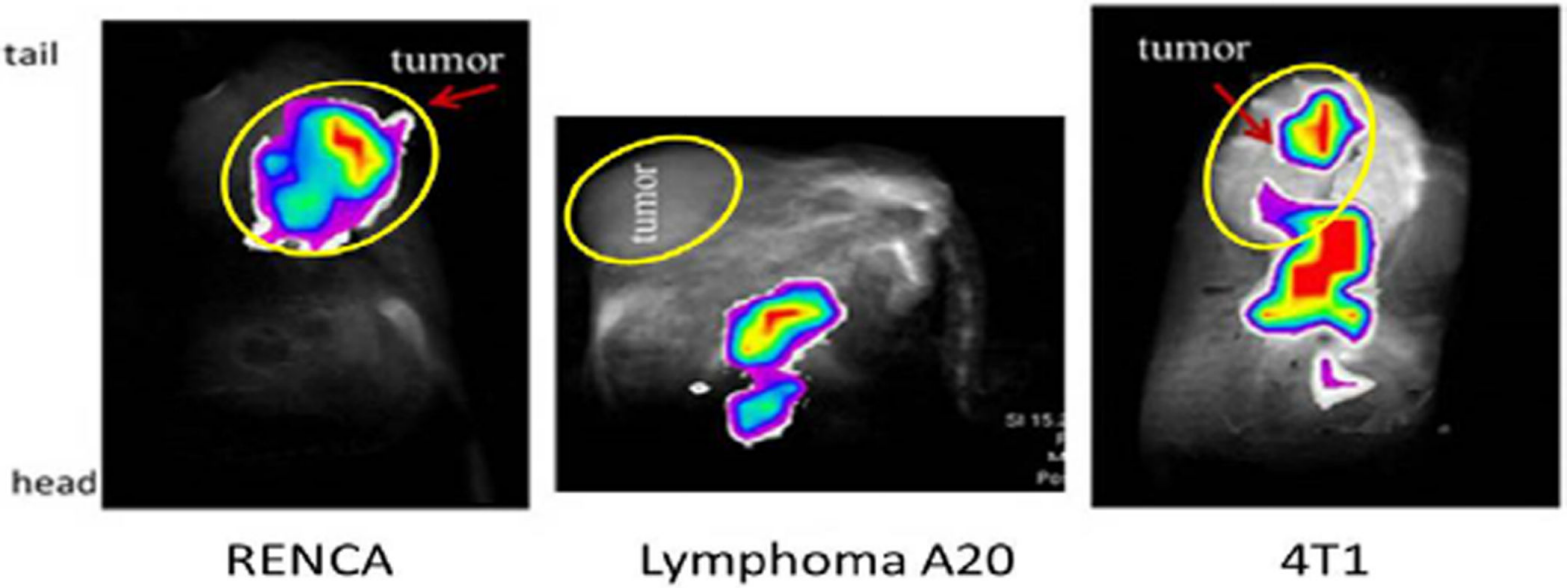
*In vivo* (iv. injection) ^13^C imaging in three subcutaneous cancer animal models: The carbon-13 images (false color) illustrate the difference in the biodistribution of hyperpolarized DES after intravenous injection of 20 µmol of contrast agent in three types of allograft tumor animal models: RENCA, lymphoma A20, and breast 4T1. Tumors are circled in yellow and labeled. In all five RENCA tumor bearing animals, the majority of the ^13^C signal is seen within the tumor (as seen above) or at edges of the tumor. In seven out of eight lymphoma A20 tumor bearing animals, the majority of signal is seen outside of tumor in the main body of the animal similar to the image above. The biodistribution of the compound was more mixed in the six 4T1 breast tumor bearing animals. In 50% of the animals, signal is detected at the edges of the tumor (as in image above) while the other half of the animals no signal is detected in the tumor. In summary, ten to thirty seconds after injection RENCA tumors take up the majority of hyperpolarized DES while lymphoma A20 and 4T1 breast tumors have significantly reduced uptake.

The biodistribution of DES was observed through the use of carbon-13 imaging. 20 µmol of hyperpolarized DES was injected via the tail vein. An overlay of the carbon-13 image with the proton image of the same geometry was created. Figure 5 and Table 2 illustrate the difference in the biodistribution of hyperpolarized DES in three types of allograft tumor animal models: 4T1, lymphoma A20, and RENCA. In all five RENCA tumor bearing animals, the majority of the ^13^C signal is seen within the tumor as seen in Figure 5 or at edges of the tumor. In seven out of eight lymphoma A20 tumor bearing animals, the majority of signal is seen outside of tumor in the main body of the animal. The biodistribution of the compound was more mixed in the six 4T1 breast tumor bearing animals. In 50% of the animals, little to no signal was seen in the tumor while in the other half of the animals signal was detected in and at the edges of the tumor. In summary, ten to thirty seconds after injection RENCA tumors take up the majority of hyperpolarized DES. This increased uptake of DES in RENCA and 4T1 cells was also observed in tissue culture.

**Figure 6:**
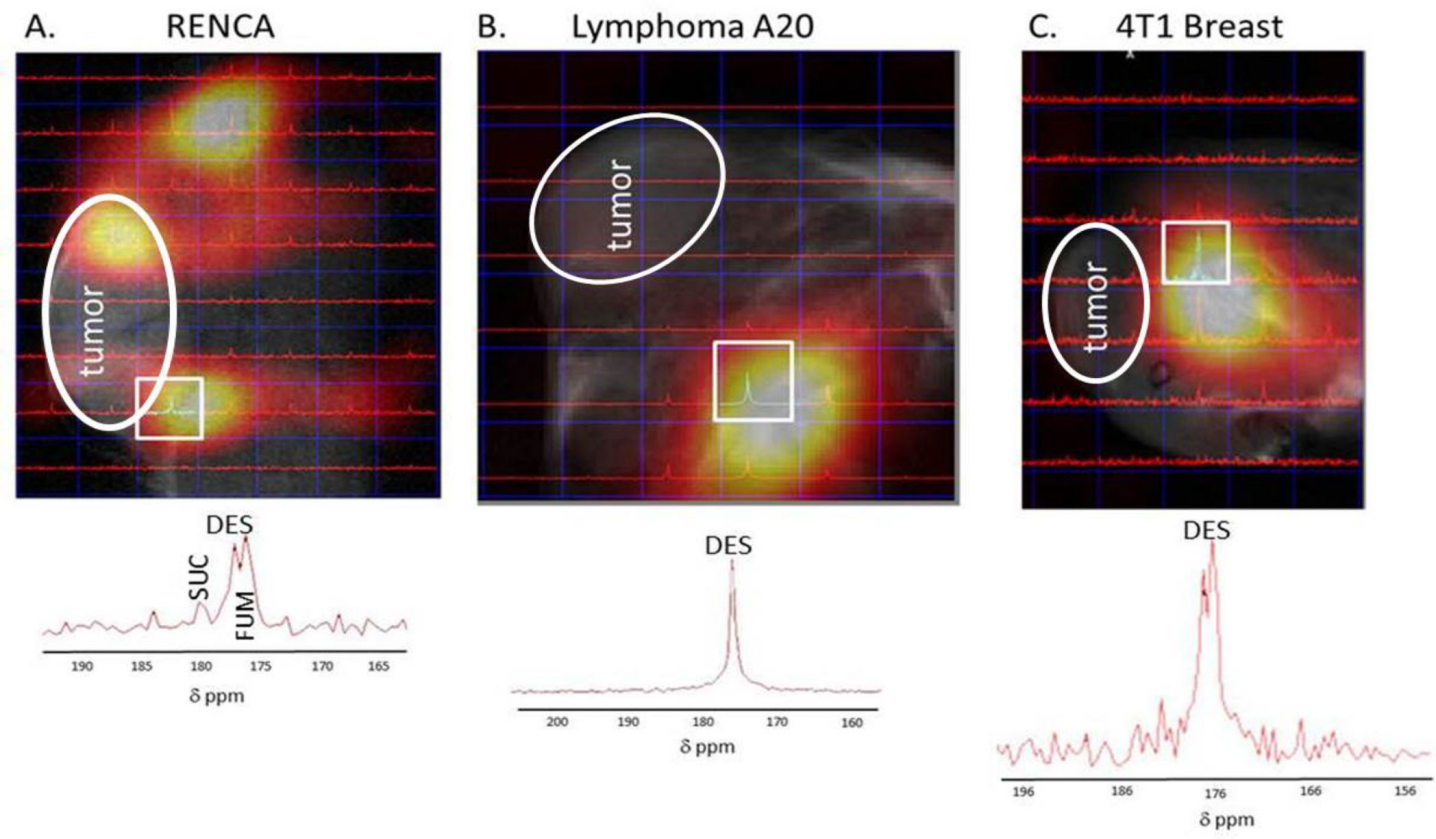
DES CSI: Representative ^13^C CSI from tumor bearing animals after intravenous injection of 20 µmol of hyperpolarized diethyl succinate. Tumors are labeled and circled for clarity. **A**. In RENCA tumor bearing animals, CSI reveals pronounced uptake in the tumor and some metabolically active areas. The image on the top is the heat map generated from the CSI in a RENCA tumor bearing animal and the highlighted voxel’s spectrum is below the image. **B**. In lymphoma A20 tumor bearing animals, CSI reveals little uptake and metabolism of hyperpolarized DES. The spectrum from the voxel with the majority of signal can be seen in more detail below the heat map and only one peak for DES is seen. **C**. The heat map generated from the CSI in a 4T1 breast tumor bearing animal reveals little tumor uptake but metabolism is seen in some of the voxels within the CSI matrix.

**Figure 7:**
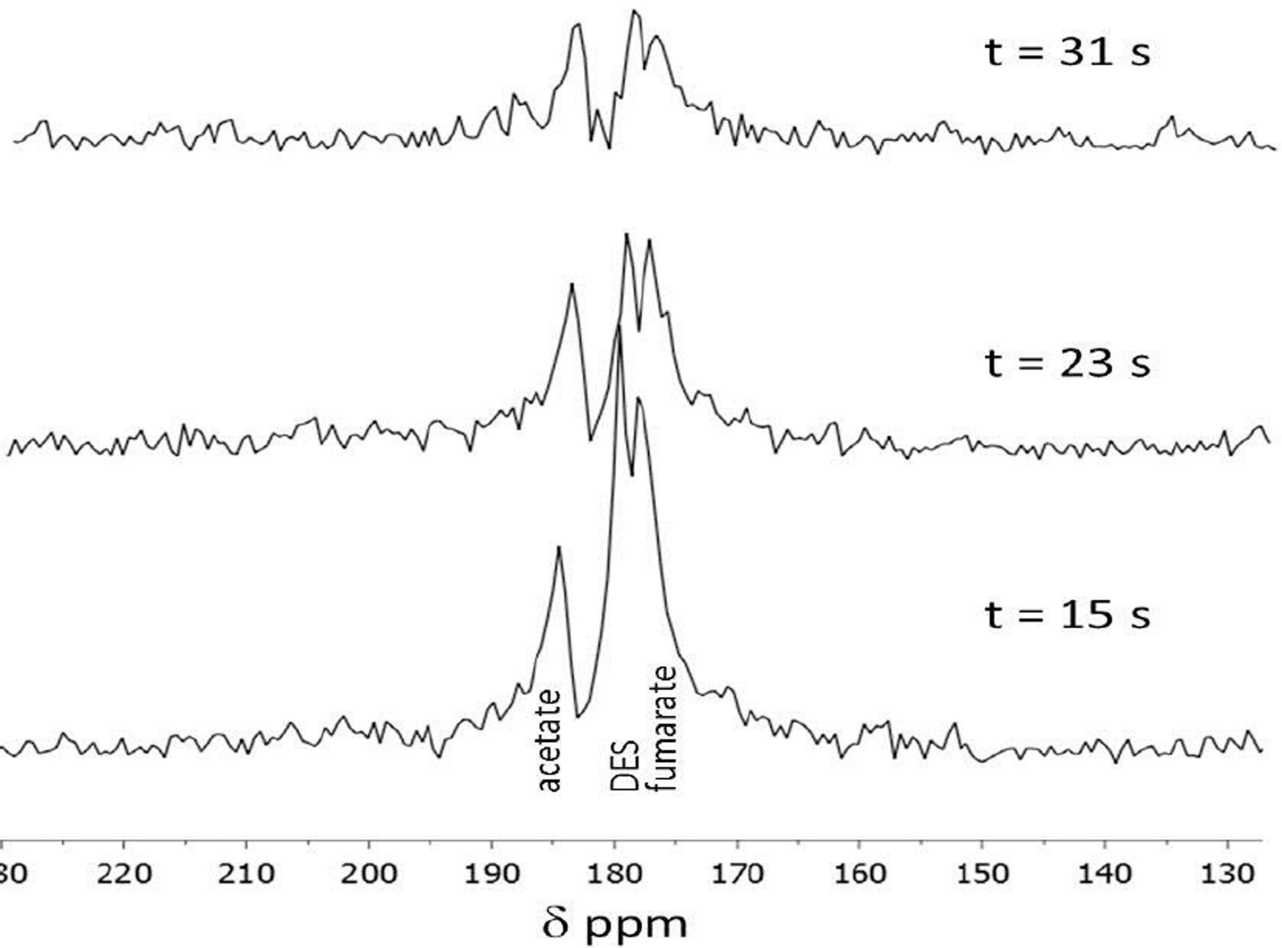
Representative spectroscopy of hyperpolarized DES in a RENCA bearing animal. The first spectrum is taken 30 s after tail vein injection of 10 µmol of DES and uses a 30° flip angle. The tumor is placed in the center of solenoid coil and a small ^13^C-acetate phantom was placed next to the tumor as a reference.

We further analyzed the metabolism of hyperpolarized DES within and around the tumor using chemical shift imaging. Using ^13^C CSI, spectroscopic data specific to the tumor can be observed. Our CSI allows for a group of voxels (8×8 or 12×12) in a two dimensional grid to be placed over the tumor. The CSI spectroscopy grid can then be correlated to the ^1^H MRI image taken with the same placement (Figure 6). Our CSI revealed that hyperpolarized DES is taken up and metabolized differently in the three cancer models (RENCA, Lymphoma A20, 4T1) (Figure 6). In RENCA tumor bearing animals, CSI revealed pronounced uptake in the tumor and some metabolically active areas. The heat map generated from the CSI in RENCA tumor bearing animals confirms the ^13^C FISP imaging result. In lymphoma A20 tumor bearing animals, CSI reveals little uptake and metabolism of hyperpolarized DES. The spectrum from the voxel with the majority of signal can be seen in more detail below the heat map and only one peak for DES is seen. The heat map generated from the CSI in a 4T1 breast tumor bearing animal reveals little tumor uptake but metabolism is seen in some of the voxels within the CSI matrix. Our *in vitro* assays, expands on our understanding of DES metabolism (see next section). To further analyze the metabolism of DES in 4T1 and RENCA tumor bearing animals, a solenoid coil with an active region of ~ 4 cm was used to determine the metabolism of the compound more thoroughly (Figure 7). Non-selective spectroscopy was done and metabolism was seen in all RENCA tumor bearing animals.

### *In vitro* assays of DES

*In vitro* data provided crucial information about how DES was metabolized. A series of labeled 1,4-^13^C diethyl succinate studies with RENCA, 4T1, lymphoma A20, and PC3M cell lines were conducted to delineate the metabolic profiles of DES metabolism across a series of cancer cell lines. Esterase activity was observed when fetal bovine serum (FBS) was added to the growth media. Complete conversion of 3.5 mM DES to succinate was observed in 1 hr when growth media contained 10% FBS. RENCA, 4T1, and PC3M cells DES studies were performed in RPMI-1640 media without FBS. No breakdown products (n = 3). On the contrary, no significant uptake of DES in lymphoma A20 incubations (n = 2) was detected. DES uptake experiments were performed by taking 200 µl aliquots of the growth media initially (t = 0) and at different time points. The integration of the DES peak was used to determine the amount of DES being taken up by cells. Variable of 3.5 mM DES were found in RPMI-1640 media after 6 hours and very little (< 1%) was seen when 3.5 mM DES is incubated in spent media (media taken after cell growth for 6 hours). Significant conversion of 3.5 mM DES to monoethyl succinate (MES) and succinate were observed only when intact RENCA or 4T1 cells are included in the incubation results were observed with changes in labeled diethyl succinate (3.5 mM) in the media from RENCA and 4T1 cells. In some experiments, we observe DES uptake percentages as high 32 - 20% in 1 hour while in others we see little uptake in 1 hour or the uptake decreases over time. This variability could be partially due to that the hydrophobicity of DES allowing diffusion in both directions (in and out of the cell) with no specificity. Furthermore, the same variability occurred when uptake experiments were performed with 1 mM 1-^13^C pyruvate incubations with RENCA and 4T1 cells (n = 3). Based on these results, we believe the inconsistencies in DES uptake percentage are specific to the RENCA and 4T1 cell type and not the compound itself. Preliminary results with PC3M (prostate cancer) cells incubated with 1 mM 1-^13^C pyruvate or 1 mM diethyl 1,4-^13^C succinate have shown improved uptake of pyruvate (20%) and DES (26%) compared to RENCA and 4T1 cell lines. To confirm that DES was being taken up by cells, high resolution ^13^C spectroscopy experiments were performed on homogenates of cells pellets of 4T1 and RENCA incubated and not incubated with DES. Several resonances within the carbonyl region (184 – 175 ppm) were seen in the carbon-13 spectroscopy of DES(+) incubations and not in controls. In addition, after a 8 hour incubation with DES the media was removed, lyophilized and the carbon-13 spectra was taken. Higher concentrations of carbon-13 labeled fumarate and lactate were seen with RENCA cells incubated with DES. With 4T1, higher concentrations of carbon-13 labeled lactate were seen in the media incubated with DES (Table 3).

## Discussion

Our laboratory has been working on developing a broad utility Krebs cycle metabolic imaging agent for several years now [19,20,23]. Using PHIP polarization, we were able to hyperpolarize deuterated and esterified versions of succinate. In this work, the uptake and metabolic flux of these agents in five cancer allograft models: breast 4T1, renal cell carcinoma RENCA, colon CT26, lymphoma NSO, and lymphoma A20 has been determined. With hyperpolarized SUC, limited metabolism was observed in lymphoma A20 while multiple metabolites of SUC were observed in RENCA tumors. In RENCA, a relatively high turnover rate of hyperpolarized SUC was estimated. Even though precise quantification methods need to be further refined, PHIP provides a minimum estimate of Krebs cycle rate in RENCA and lymphoma A20 tumor models. It was estimated that net flux through the portion of Krebs cycle described by the metabolic products were ~5 µmoles/minute/gram tumor in RENCA and 0.7 µmoles/minute/gram tumor in lymphoma A20. It is likely that these are minimum estimates of (partial) Krebs cycle rates limited by a number of factors including substrate delivery and local concentrations which are below or close to enzyme K_m_ (K_m_ of Fumarase: 1.3 ×10^−3^ M; K_m_ of Citrate Synthase: 5.3 × 10^−5^ M). Furthermore, a unique feature of hyperpolarization (a fast T_1_; T_1_~40–100 seconds) implies that the data presented here will largely be defined by “initial reaction rates” over the first 40–100 seconds of exposure of rate limiting Krebs cycle enzymes to their substrate(s).

For hyperpolarized DES, more pronounced uptake occurs in RENCA tumors versus lymphoma A20 or 4T1 breast tumors. Different metabolic profiles of DES were observed in the three different animal cancer models using ^13^C CSI. No metabolic flux of DES in lymphoma A20 was observed. This could be due to differences in the compounds, the pH of the media used for SUC versus DES injections, or the increased sensitivity from using a surface coil in the hyperpolarized SUC experiments compared to solenoid and volume coil in DES experiments. However, CSI spectroscopy revealed DES to be readily metabolized in RENCA and 4T1 breast tumors. This was validated through *in vitro* incubation studies of DES. Voxels within the CSI images were centered to 176.4 ppm corresponding to DES. Based on the spectroscopy, voxels with metabolic products in RENCA and 4T1 animals reveal three to five resonances. Strong resonances occur downfield at 178 ppm and up field of DES at 173 ppm. Resonances at 179 ppm and 184 ppm are also seen within some of the CSI voxels. Due to the broad line widths we achieved in our *in vivo* spectroscopy, it is hard to determine the exact compound each resonance corresponds to. Another group has reported the use of hyperpolarized diethyl succinate for metabolic imaging of the heart [24]. They concluded that DES was metabolizing into monoethyl succinate (MES) with two resonances corresponding to the free carboxylic acid at 183.8 ppm and the remaining ester carbonyl at 178.8 ppm referenced to DSS. In our spectroscopy results, we do not see equivalent resonances for the breakdown of DES to MES in all voxels. However, some of the *in vivo* resonances could correspond to MES but due to the broad line widths and close chemical shifts for carbonyls of Krebs cycle metabolites our spectroscopy is not definitive. In RENCA tumors and cells, we confirmed the metabolic conversion of DES to fumarate using a solenoid coil and *in vitro* DES incubation studies.

Based on our media and cell pellet studies, we do observe cellular uptake and conversion of DES in our *in vitro* assays in RENCA and 4T1 cells but not in Lymphoma A20 cells. The level of DES in the media was highly variable over time but behaved in a similar manner to pyruvate incubations. Our cell pellet data revealed a number of carbon-13 labeled metabolites and lyophilized media samples revealed significant increases in lactate and fumarate in the DES (+) incubations versus the control experiments DES (−). Based on the lactate values, DES is metabolized by RENCA approximately three times greater than in 4T1 cells. These results along with data from other research laboratories [18– 20] reveal an interesting caveat in hyperpolarization studies, because the sensitivity enhancement is so high with hyperpolarized MR, only a small percentage of labeled compound [25–27] needs to be converted for a signal to be observed.

In summary, reproducible uptake and metabolism of SUC and DES was observed in RENCA tumor bearing animals but not the other cancer models. Our results underscore the role of cellular uptake for imaging compounds and the heterogeneity of cancer cells. The increased uptake of DES and SUC in RENCA tumors is probably due to the cells being derived from renal proximal convoluted tubular (PCT) cells. PCT cells are densely packed with mitochondria and are known to reabsorb Krebs cycle metabolites (citrate and succinate) [28,29]. Our results are similar to classical biochemical and ^14^C succinate studies that demonstrated succinate to be readily taken up by renal cells [28,29]. We are currently determining if other succinate derivatives (such as benzyl esters) would have broader and better cellular uptake than SUC and DES.

## Supplementary Material

Suppl methods

## Figures and Tables

**Table 1:** Number of in vivo hyperpolarized SUC experiments per concentration and pH.

Type of Tumor	*Total Exps	Number of Animals	SUC 10–20 mM	SUC 30–60 mM	SUC pH 3–4.5	SUC pH 8.5–9
Colon 26	29	26	26	0	5	21
Lymphoma A20	9	9	9	0	7	2
Breast 4T1	17	14	10	7	3	14
RENCA	16	14	16	0	15	1
Lymphoma NSO	11	8	11	0	10	1

* Total of number of experiments is higher than number of animals due to multiple injections.

**Table 2:** Number of *in vivo* hyperpolarized DES experiments with description of biodistribution and metabolism.

Type of Tumor	Total Number of Animals	Majority of signal in tumor or edges of tumor	Majority of signal not in tumor	Metabolism Seen
RENCA	5	5	0	5
Lymphoma A20	8	1	7	3
Breast 4T1	6	3	3	6

**Table 3:** Summary of *in vitro* 1,4-^13^C Diethyl Succinate (DES) experiments.

Cells	Average DES uptake in 1 hr*	^13^C fumarate in media**	^13^C lactate in media**
RENCA	4 % (n = 3)	1.2 ± 0.2 (+DES) 0.44 ± 0.09 (−DES) P = 0.005	9.2 ± 2.7 (+ DES) 4.7 ± 0.58 (−DES) P = 0.05
Breast 4T1	4 % (n = 3)		2.9 ± 0.5 (+DES) 1.8 ± 0.2 (−DES) P = 0.02
Lymphoma A20	0% (n = 2)		

* The DES uptake experiments for RENCA and 4T1 cell line were highly variable from 32–22% to 0% after a one hour incubation.

** Normalized concentration of fumarate and lactate in lyophilized media after 8 hour incubation with DES (+) or without DES (−). In addition, the difference between ^13^C lactate (DES +) values between RENCA and 4T1 is significant (P < 0.02).
