# Supplementary material for: Towards Real-time Metabolic Profiling of Cancer with Hyperpolarized Succinate
Source: J Mol Imaging Dyn. Author manuscript; Available in PMC 2016 Aug 18. (PMC4989923; doi:10.4172/2155-9937.1000123)
Supplement: Suppl methods [file NIHMS804581-supplement-Suppl_methods.docx]

**Supplemental Methods and Figures for “Towards Real-Time Metabolic Profiling of Cancer with Hyperpolarized Succinate”**

1. **Animals:** Female BALB/c mice (6 – 8 weeks old), purchased from Harlan S/D, were implanted with 5x10^6^ cells from one of five tumor cell lines: CT26 colon, 4T1 breast, NSO lymphoma, lymphoma A20 or renal carcinoma RENCA cell line. Tumors developed on the dorsum subcutaneously and were allowed to grow >0.5 cm diameter before experimentation. Each mouse was anesthetized with 1.5% isoflurane gas and 0.8 L/min oxygen by face mask. The lateral tail vein was catheterized with 30-gauge tubing (MVT-1, Braintree Scientific), attached to a two-foot PE50 extension. A warm-water tail bath produced the vasodilation critical to successful tail vein cannulation. The sedated mouse was then placed in a heated cradle within the bore of a 4.7T MR scanner (Bruker Paravision). Hyperpolarized SUC was injected into tumor bearing mice with CT26, 4T1, NSO, A20, or RENCA cells lines. DES was injected into mice bearing 4T1, A20 or RENCA. MRS and MRI scans were then performed. All animal experiments were approved by the IACUC of Huntington Medical Research Institutes.
2. **Other MR Imaging and Spectroscopy Sequences Utilized**

**2.1** **Hyperpolarized Succinate studies**

Additional carbon MRI images were acquired with a two turn 25 mm diameter dual resonance loop coil in a 4.7T Bruker Scanner. The loop was positioned around the tumor. A 3M sodium acetate (Cambridge isotopes, 99% ^13^C enrichment at C1) marker was placed on top of the tumor and used for co-registration of the 13-carbon and proton images. The center frequencies for the images are set at the C1 acetate and water resonances, respectively. A one centimeter axial slice was acquired through the center of the tumor with a 6 cm X 6 cm field of view with a spin-echo sequence with TE = 14.6 ms, TR = 220 ms and AVG = 2. A 128 X 128 matrix was acquired in 56 seconds with an in-plane resolution of 0.469 mm X 0.489 mm. A 13-carbon gradient echo image was acquired in the same location and field of view as the proton immediately after injection of 0.5 mL of 10-20 mM hyperpolarized succinate. A 32 X 32 matrix was used with the read acquisition zero filled to 64; an in-plane resolution of 0.938 mm X 1.875 mm was obtained. A pulse angle of 55 degrees was used for each phase acquisition with the total time of 3 seconds; TE=7.9 ms, TR = 96 ms. ^13^C images were co-registered with proton images, based upon the fixed acetate-^13^C phantom, so that the precise distribution of hyperpolarized signal can be determined in each animal.

Hyperpolarized ^13^C was unevenly distributed throughout the tumor volume. To better correlate spatial and metabolic information ^13^C - chemical shift imaging were performed on some RENCA and lymphoma A20 tumors using a variable angle fast gradient echo sequence. 3.5 seconds was required to acquire a matrix of 16 X 16 with a field of view of 6 X 6 cm. The in-plane resolution was 3.75 mm squared with a slice thickness of 1 cm, sufficient to encompass a majority of the tumor with the previously described dual tuned transmit and receive surface coil.

**2.2 Hyperpolarized Diethyl Succinate Studies**

To further analyze metabolic data two RENCA and 4T1 tumor bearing animals were placed in a ^1^H/^13^C solenoid coil [[15](#_ENREF_15)] with the subcutaneous tumor placed in the middle of the active area of the coil. After injection of hyperpolarized DES, consecutive ^13^C MRS was acquired using a pulse and acquire approach using a non-selective Gaussian radio frequency pulse for excitation and using usually a pulse angle of 20-30^o^ (bandwidth 25000 Hz and acquisition size 2048) every 7-8 seconds for about 1 minute after injection of hyperpolarized diethyl succinate into the mouse.

1. **Diethyl Succinate Tissue Culture Experiments**
   1. **Tissue Culture**

4T1 (CRL-2539), RENCA (CRL-2497), and A20 (TIB-208) cells were purchased from ATCC. PC3M cells were purchased from the CCSG-funded characterized cell line core facility at M.D. Anderson. All cell lines were grown in RPMI-1640 medium supplemented with 10% FBS (Sigma), 100units/mL penicillin, and 100µg/mL streptomycin (Cellgro). In addition, the RENCA cells were supplemented with 1 mM pyruvate (Lonza), 1X MEM non-essential amino acids (Cellgro), and 2 mM glutamine (Cellgro); and the A20 cells were supplemented with 1 mM pyruvate, 2.38 g/L HEPES (Sigma), 2.5 g/L glucose (Sigma), and 50 µM β-mercaptoethanol. When necessary, non-pre-sterilized solutions were sterilized by filtering through a 0.22 µm filter. As esterases present in the FBS contributed toward significant breakdown of the double-carboxyl-labeled ^13^C-diethylsuccinate (^13^C-DES), all uptake and incubation experiments, when possible, were performed in media that had not been supplemented with FBS, although all other components were still added. As A20 cells had significantly reduced viability when grown in the absence of FBS, uptake and incubation experiments with DES were performed in the presence of 1% FBS which allowed for cells to be viable but prevented esterase-mediated breakdown of ^13^C-DES. For each incubation, multiple samples of ~ 2 x 10^6^ cells were incubated for six hours at 37ºC with 5 mL of incubation media supplemented with 3.5 mM 1,4-^13^C-DES. 50 µL aliquots were taken at time points of 0, 1, 2, 3, 4, and 6 hours with the volume from multiple samples pooled and stored at -80ºC. After each experiment, the cells were counted with trypan blue (0.4% in 0.81% NaCl and 0.06% K_2_HPO_4_), spun down at 130g with a centrifuge, and the pellet stored at -80ºC.

- 1. **Homogenization:**

Cell pellets were treated with 3 mL of ice-cooled 2:1 methanol to water solution and ~ 500 µl of MP Biomedicals lysing matrix D beads. Samples were put through a freeze/thaw cycle three times with a one minute vortex performed after each cycle. The homogenates were then centrifuged for 10 min at 4000 RPM, supernatant was removed and lyophilized overnight, and the remaining metabolites were dissolved in deuterium oxide and NMR standard with buffer was added (final concentration 1-5 mM DSS sodium salt (Sigma-Aldrich, 178837), 50 mM potassium phosphate pH 7.5).

- 1. **NMR Spectroscopy of Cell Culture Experiments:**

All ^1^H- and ^13^C-NMR spectra were taken utilizing a Bruker Avance III HD 500 MHz spectrometer with a Prodigy BBO cryoprobe. To determine the extent of cellular ^13^C-DES uptake, ^13^C-NMR spectra of each of the pooled media samples taken at different time points were collected using a 30^o^ pulse, a relaxation delay of 6 seconds, 128 scans, a spectral width (SW) of 29760 Hz, and referenced to DSS at 0.00 ppm; ^1^H-spectra used a relaxation delay of 6 s, 16 scans, a SW of 10245 Hz and were referenced to DSS at 0.00 ppm. Water suppression was performed with presaturation (Bruker Water Sequence). The ^13^C-NMR spectra of each of the extracted cell pellet samples were collected using a relaxation delay of 6 seconds, 2560 scans, a spectral width (SW) of 29760 Hz, and referenced to DSS at 0.00 ppm. ^1^H-spectra used a 30^o^ pulse, relaxation delay of 6 s, 128 scans, a SW of 10245 Hz and were referenced to DSS at 0.00 ppm. Data was analyzed in MestraNova or TopSpin2.1 (line broadening 0.5 Hz) baseline corrected and phased manually. The concentrations of different metabolites were determined by taking the ratio of the integrated value of the resonance for the metabolite (such as lactate) over the integrated value of the DSS standard. These values were then used to measure the amount of DES converted in cell culture. Student t-tests were performed using GraphPad Prism for DES (+) and DES (-) feeding studies.

**Supplemental Figure 1**


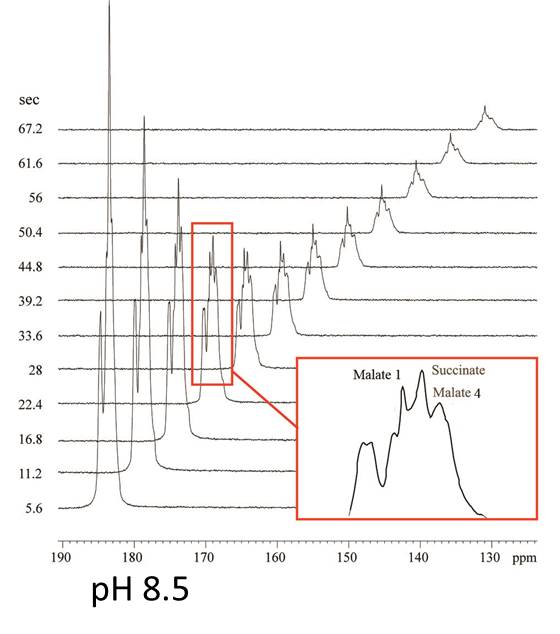


Hyperpolarized metabolic products of hyperpolarized SUC at pH 8.5 were detected in the Lymphoma A20 tumor 10 seconds after injection via the tail vein and persisted for 67 seconds. The metabolites are identical to the experiment performed at pH 3 – 4.5 (Figure 3A). Note the chemical shift change of intra-tumoral succinate resonance from 180 to 183 ppm and preponderance of C1 over C4 malate within tumor injected with hyperpolarized ^13^C succinate generated under alkaline condition.
